# Associations between Adherence to Combinations of 24-h Movement Guidelines and Overweight and Obesity in Japanese Preschool Children

**DOI:** 10.3390/ijerph17249320

**Published:** 2020-12-13

**Authors:** Hyunshik Kim, Jiameng Ma, Kenji Harada, Sunkyoung Lee, Ying Gu

**Affiliations:** 1 Faculty of Physical Education, Sendai University, Miyagi 9891693, Japan; hs-kim@sendai-u.ac.jp (H.K.); jm-ma@sendai-u.ac.jp (J.M.); kn-harada@sendai-u.ac.jp (K.H.); 2 Advanced Research Center for Human Sciences, Waseda University, Saitama 3591192, Japan; 3 Department of Life Physical Education, Myongji University, Seoul 03674, Korea; iamlsk@mju.ac.kr; 4 College of Sports Science, Shenyang Normal University, Shenyang 110034, China

**Keywords:** accelerometer, physical activity, screen time, sleep duration, adiposity, preschool children

## Abstract

The interactions between movement behaviors (physical activity, screen time, and sleep) affect the health of preschool children. Therefore, we examined the status of adherence to combinations of 24-hour movement guidelines (24-h MG) in Japanese preschool children and determined the associations between overweight/obesity and adherence to these 24-h MG. This cross-sectional study was conducted with 421 children aged 3–5 years (216 boys and 199 girls) living in the northeastern region of Japan. To evaluate the 24-h MG, physical activity over one week was measured using a three-axis accelerometer. For screen time and sleep duration, a questionnaire survey was conducted. Children who failed to meet all the 24-h MG had a higher probability of overweight/obesity than those who met all the 24-h MG (odds ratio 1.139, 95% confidence interval: 1.009, 1.285). The percentage of adherence to the 24-h MG was 91.6% for physical activity, 82.5% for sleep duration, and 33.7% for screen time, and only 21.5% of the children adhered to all three areas of the guidelines. Our findings have important implications for developing public health policies and effective intervention programs for preschool children.

## 1. Introduction

Early childhood is a time of rapid physical and mental development during which time a child’s lifestyle habits are formed and changes and adaptations are made to the environment. During this period, regular and sufficient levels of physical activity are critically important for health, efforts should be made to limit screen time, and sufficient sleep duration should be ensured [1].

Previous studies have typically adopted separate approaches for investigating the lack of physical activity (PA), screen time, and insufficient sleep of preschool children, and each of these has been independently considered as a health risk factor [2]. According to systematic reviews of preschool children, regular PA is associated with improved motor skills, cognitive development, psychosocial development, and cardiac metabolic health [3,4]. Screen time refers to the time spent on screen-based behaviors; in children with long periods of screen time, the ratio of overweight and obesity and high-density lipoprotein-cholesterol levels were high, fitness levels were low, and there were lower educational attainments and poor sleep outcomes [5,6]. Moreover, insufficient sleep duration is reportedly associated with an increased risk of obesity, decreased emotional regulation, decreased academic success, and degraded quality of life [7,8].

Until recent years, the association between the time spent on each of these behaviors and health outcomes has traditionally been investigated separately or only as partial adjustments to the time spent on other behaviors [9]. However, to maintain and promote the optimal health status of preschool children, it is important to increase PA, reduce screen time, and ensure sufficient sleep duration within each 24-h period. There has been increasing interest in obtaining strong evidence supporting the interaction between these movement-related behaviors over 24-h and the effect on health [10,11,12].

Recently, “24-Hour Movement Guidelines” were developed in Canada [13] and published by the World Health Organization (WHO) [14]. These guidelines involve a transition from a single behavior to integrated behaviors, representing a paradigm shift regarding movement behavior. For example, even if the recommended daily PA level (at least 60 min of moderate-to-vigorous intensity exercise out of at least 180 min of PA) is met, a long screen time and short sleep duration will reduce the health benefits of PA. As a result, since the time spent on one behavior affects other behaviors for the remainder of the day, and the composition and combination of movement behaviors are interdependent, the combination of movement behaviors within a 24-h period has an important health effect on preschool children [15,16]. Each of these behaviors is repeated and habituated on a daily basis, and since these can potentially become lifestyle habits for preschool children, it is pivotal that all three types of movement behaviors (PA, screen time, and sleep) over 24-h are considered in relation to health.

It is estimated that 6.7% of children under the age of five are overweight or obese worldwide, and this ratio was expected to reach 9.1% by 2020 [17]. In addition, according to a WHO report, in 2016, more than 41 million children under five years of age were overweight or obese, and nearly three-quarters of them lived in Asia and Africa [18]. Likewise, in recent years in Japan, overweight and obesity in early childhood have become major public health concerns [19]. In Japan, PA guidelines are available for preschool children; however, no guidelines comprehensively consider PA, screen time, and sleep [20]. Therefore, for more effective prevention of the current crisis of child obesity, evidence supporting a broader, more comprehensive, and integrated approach to the understanding and promotion of 24-h movement behavior for preschool children is required [21].

The purpose of this study was to investigate adherence to the new 24-hour movement guidelines (24-h MG) for Japanese preschool children and to determine whether adherence to the different movement behaviors outlined in the 24-h MG is associated with adiposity.

## 2. Materials and Methods

### 2.1. Study Design and Participants

This was a cross-sectional study, and for the study data, convenience samples were collected from six nurseries among daycare centers located in the northeastern region of Japan that volunteered to participate in “the Study on the Improvement of Life Habits for Children in East Asia.” Preschool children aged 3–5 years who were in good overall health with no physical or mental disorders were selected. In addition, prior to participating in the study, a written guide on the study and a consent form were provided to the participating children and guardians, and the subjects who gave their consent to participate were included in the analysis.

For preschool children from 6 nurseries (*n* = 473) located in Miyagi Prefecture in the northeastern region of Japan, accelerometer measurements were taken, and questionnaire surveys were conducted between October and November 2018. Accelerator measurements and surveys on movement behaviors were conducted in the same week. Data from 421 subjects (51.9% boys; 48.1% girls) were analyzed after subtracting data without consent from the study (*n* = 28); the criterion for accelerometer measurement was data from devices not being worn for more than 600 min a day and more than four days a week (*n* = 16) and data with errors such as an incomplete questionnaire.

The study received prior approval from the Sendai University Ethics Committee, Faculty of Sports Science (SU29-22).

### 2.2. Measurements

#### 2.2.1. Physical Activity

The amount of PA, a triaxial accelerometer (Active Style Pro HJA-750C, Omron Health Care Co., Ltd., Kyoto, Japan) was used. The Active Style Pro provides metabolic equivalent of task (MET) values derived from predictive equations for adults; however, it has been reported that MET values result in overestimated results for children compared to adults [22,23].

Therefore, we used the following conversion equations (1) and (2) obtained from the results of previous studies:

Ambulatory activities: 0.6237 × the value of MET by the Active style Pro + 0.2411(1)

Nonambulatory activities: 0.6145 × the value of MET by the Active style Pro + 0.5573(2)

The participants were asked to wear an accelerometer on their waist for 1 week from the time that they woke up until they went to sleep (7:00 and 21:00), except when they were taking a shower or swimming. If the accelerometer value remained at 0 for 20 min or longer, then it was assumed that the participant was not wearing the accelerometer. In terms of PA, the triaxial accelerometer measured sedentary time (≤1.5 metabolic equivalents (METs)), light-intensity (1.6–2.9 METs), and moderate and vigorous PA (3 METs or above), and these measures were evaluated every 10 s. To measure PA per day, the data were extracted when the participants wore the accelerometer for 600 min or more per day, over a period of 4 days per week [24].

#### 2.2.2. Screen Time

Screen time refers to the time spent on screen-based behaviors, includeing recreational screen time, stationary screen time, sedentary screen time, and active screen time [25]. Screen time was assessed by asking parents how much time their children spent watching TV/video and using smartphones/tablets in the past week using the following questions: (1) how many media devices, including TVs, smartphones, tablets, and computers, are available at home? (2) how much time on average does your child spend on a day watching TV or videos? and (3) for how long on average in a day does your child use electronic devices, such as smartphones, tablets, and computers? Subsequently, parents were asked to indicate the average number of days per week and weekends that their child spent on-screen-viewing time base on six options: 0, 1–29, 30–59, 60–119, 120–179, or ≥180 min. The parents also provided the daily average screen time on weekends and weekdays in written answers [26]. To calculate the average time spent on the screen-viewing activities per week, the number of days per week or weekend the child spent time on activities was multiplied by the mid-category values of the duration of the activity per day. Subsequently, the child’s average daily screen time was calculated (average daily screen time = (weekday screen time × 5) + (weekend screen time × 2)/7).

#### 2.2.3. Sleep Duration

For sleep duration, questions such as “how many hours does a child normally sleep at night on weekdays?” and “how many hours does a child normally sleep on weekends?” were surveyed. We asked the parents to record the children’s sleep duration, including naps, on weekends. In addition, for nap time at the nursery, the childcare teachers were asked to record the nap time on weekdays, and the daily sleep duration was calculated as the sum of the durations of night sleep and daytime nap time. Given that sleep duration is always included in the times children spend in the nurseries, we measured the children’s sleep duration by recording the children’s sleep duration from Mondays to Fridays. The total sleep duration was calculated as ((weekday sleep duration × 5) + (weekend sleep duration × 2)/7).

#### 2.2.4. Body Mass Index

The height and weight of preschool children were measured in units of 0.1 cm and 0.1 kg, respectively, and objective measurements were made by the researchers. The BMI z-score was calculated according to the WHO growth criteria [27].

For participants 5 years and below, overweight and obesity were classified as BMI z-score above 2 standard deviation and above 3 standard deviation, respectively. For participants over 5 years, overweight and obesity were classified as BMI z-score above 1 standard deviation and above 2 standard deviation, respectively [28]. Participants were then categorized into two groups: normal weight and overweight/obese groups.

#### 2.2.5. Adherence to the 24-h Movement Guidelines

The following recommendations were used to evaluate the new 24-h MG for preschool children [14]: PA guidelines, 180 min of total PA including 60 min/day of moderate to vigorous PA; screen time guidelines, less than 1 h per day; and sleep duration guidelines, 10–13 h within 24 h.

### 2.3. Statistical Analysis

Frequency analyses were conducted to investigate the percentage of preschool children who met the PA guidelines, screen time guidelines, and sleep guidelines or combinations of these guidelines (PA + screen time, PA + sleep duration, screen time + sleep duration, and PA + screen time + sleep duration). In addition, the percentage of preschoolers who adhered to the 24-h MG was calculated for each movement behavior and all combinations. Descriptive statistics were used to investigate the total duration of PA, screen time, and sleep duration for 1 week, including weekdays and weekends, and continuous variables were presented as means and standard deviations. A t-test was performed for the analysis of continuous variables (age, BMI, BMI z-scores, moderate to vigorous PA, total PA, screen time, and sleep duration), and mean values between sexes were compared. Logistic regression analyses were then conducted to examine the associations between not adhering (versus adhering) to single guidelines or combinations of each guideline and odds ratios for being overweight and obese in the sample before and after adjustment for age and sex. For the level of statistical significance, *p*-values were set to <0.05. Data analyses were performed using IBM SPSS version 25.0 (IBM, Armonk, NY, USA).

## 3. Results

Table 1 shows the descriptive statistics of preschool children participating in the study classified by sex. The mean age of the participants was 4.62 ± 0.85 years; 4.55 ± 0.86 years for boys and 4.70 ± 0.89 years for girls. Boys had significantly higher BMI values (boys 15.77 ± 1.29; girls 15.52 ± 1.26, *p* < 0.039) and BMI z-scores (boys 0.22 ± 0.96; girls 0.05 ± 0.81, *p* < 0.041) than girls. In addition, in terms of PA, moderate to vigorous PA (boys 106.82 ± 28.92 min; girls 90.31 ± 26.88 min, *p* < 0.001).

**Figure 1:**
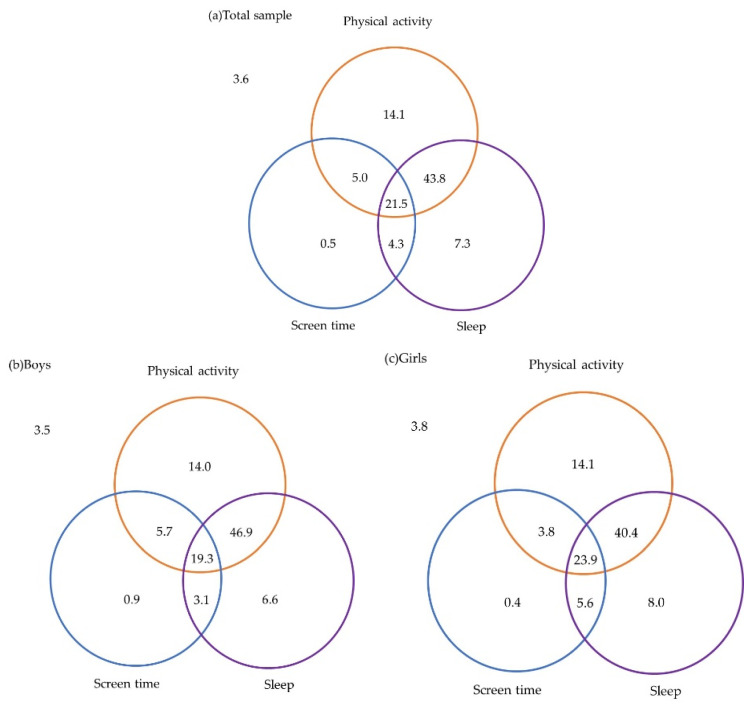
Percentages meeting 24-h movement guidelines among (**a**) overall (*n* = 421), (**b**) boys (*n* = 216) and (**c**) girls (*n* = 199) Japanese preschool children. Venn diagram: The numbers within each circle are added to the proportion of children meeting each individual guideline (i.e., 65.4% for physical activity, 88.2% for screen time and 29.5% for sleep duration). The total nonoverlap area of each circle represents the proportion of children meeting one of the three guidelines (i.e., 6.0% + 18.9% + 1.6% = 26.5%). The total overlap areas of two circles represents the proportion of children meeting two guidelines of the three guidelines (i.e., 42.9% + 1.5% + 11.4% = 55.8%). The overlap area of three circles represents the proportion of children meeting all three guidelines (i.e., 15.0%). The outside area of the circle represents the proportion of children not meeting any of the guidelines (i.e., 2.7%).

Figure 1 shows the percentage of Japanese preschool children who adhered to the 24-h MG in total and divided by sex. A total of 21.5% of preschool children met the 24-h MG, and when divided by sex, girls (23.9%) showed a higher percentage than boys (19.3%). Examining the 24-h MG combinations in more detail, 3.6% of children did not meet any of the guidelines (boys 3.5%; girls 3.8%), 21.9% (boys 21.5%; girls 22.5%) met one of the three guidelines, and 70.3% (boys 71.9%; girls 68.1%) met two of the three guidelines.

Table 2 outlines the association between the BMI z-score and 24-h MG combinations using logistic regression analysis adjusted for sex and age. Compared to children who adhered to all three 24-h MG, those who did not adhere to the 24-h MG were more likely to be overweight or obese (odds ratio 1.139, 95% confidence interval: 1.009, 1.285).

## 4. Discussion

This was the first study to examine the association between overweight/obesity and adherence to the 24-h MG in Japanese preschool children. A significant association was confirmed between overweight/obesity and adherence to 24-h MG even after adjusting for sex and age. Moreover, the key finding of this study was that 96.4% of all preschool children met one or more of the guidelines; however, only 21.5% met all three guidelines. The movement behavior that showed the highest percentage of adherence in Japanese preschool children was PA at 91.6%, followed by sleep duration at 82.5% and adherence to the guideline for screen time was the lowest at 33.7%.

For comparative analyses, we considered the results from different continents for preschool children, including Canada [1,29], Australia [30,31], the United States [32], Finland [33], Belgium [34], and Singapore [35,36] and China [37] in Asia. The percentage of adherence to all three 24-h MG was 5.5–15% in most of these previous studies; however, the results of our study with Japanese children showed slightly higher values, similar to the 24% in Finnish preschoolers [33]. We found similar rates of adherence to the PA guidelines as in American preschool children (91.5%) [32] and Australian preschool children (93.1%) [30], while the percentage of adherence to the sleep duration guidelines was similar to that of Canadian preschool children (83.9%, 82.1%) [1,29]. In addition, the percentage of adherence to the screen time guidelines was similar to that of children in Finland (35%). The results of this study were similar to those of the Finnish study [33], and the trends of high PA, long sleep duration and low screen time were similar to those reported in Canada, Australia, and the United States. Among them, studies conducted in Finland (4.7 years) [33] and Australia (4.2 years) [30] included children with a similar mean age as the children in our study (4.5 years), and they showed the most similar percentage of adherence to the 24-h MG. The Finnish study with results similar to those of the present study also used an accelerometer for objective measurement.

The rate of adherence to the PA guidelines (91.6%) in the present study was relatively higher than that in Asian countries (China and Singapore) and similar to or slightly lower than that in the United States (91.5%), Australia (93.1%), and Finland (85%). Canada [29] and Australia [30,31], which reported higher adherence rates than those in this study had a younger mean age of participants than the subjects of this study; and when compared with the study with a lower adherence rate, the age of the subjects was confirmed to be slightly higher [36,37]. The slightly different results for the percentage of adherence to PA guidelines can be partially explained by methodological differences. The use of different types of accelerometers when assessing PA or the use of different measures or cutoffs in the PA definition can affect the level of PA adherence of the children [1,30,34]. Comparing this study’s physical activity measuring device (HJA-750C) to a present study that examined the relevance of 24-h MG, obesity (68 min/day, 60.9%) [38] and fitness (67 min/day, 60.4%) [39] to elementary school students in Japan, the results were also high. In Canada [40] and the United Kingdom [41], which compare the amount of physical activity between elementary school students and youth, physical activity in youth is also high, and the reasons for this are thought to be a decrease in physical activity due to free play and educational schedule. In addition, we cannot rule out the possibility that differences in weather at the time of accelerometer measurement and in the sociocultural environment could have affected the results. This study was conducted from the end of October to the beginning of November, and this period has an average temperature of 20 °C, which may have encouraged outdoor activities in children and resulted in a slightly higher adherence rate. In addition, in previous studies, girls were reported to have lower PA levels than boys [42,43]. To improve the percentage of adherence to 24-h MG in the future, a program that considers sex differences should be employed.

The results of the present study demonstrated that 82.5% of children met the daily sleep duration guidelines of 10 to 13 h [29,32], which was similar to the findings of previous western studies from Canada and the United States; however, it differed significantly from studies conducted in China [37] and Singapore [36] in Asia (13.7%–29.5%). Although a similar adherence level to sleep guidelines was demonstrated in studies from Canada [29] and the United States [32], the mean age of the children was slightly lower (3–4.2 years). This study was conducted on children attending nurseries, and one of the characteristics of Japanese nurseries is that naps are included in the daily routine. A previous study reported that naps have a negative effect on night sleep duration [44]; however, nap time is an important part of childcare schedules in Japan and other Asian countries, and the nap time is compulsory. For a more valid international comparison of the 24-h movement behavior in western countries, the sociocultural and educational environment of Asian countries will need to be considered in future studies.

In this study, the percentage of children who met the screen time guidelines was 33.7%, which was significantly lower than that in other Asian countries (88.2% in China and 70.2% in Singapore) but higher than that in Canada (24.4%), the United States (14%), and Australia (17.3%). A low percentage of children in Japan meet the screen time guidelines. The results of this study showed a lower screen-time ratio compared to that reported in other studies, thus indicating the necessity for a program to reduce screen time for children. However, it has been reported that the use of screens for educational programs improves children’s self-esteem, social skills, and knowledge [45,46]. As such, interventions should focus on screen time that has a negative effect on free time. To further improve the findings of this study, efforts should be made to increase the adherence rate for screen time. A study that investigated screen time based on an ecological model reported that the adherence rate for screen time had a higher association with family factors such as parental modeling, the setting of TV time, and screen time of parents than with personal factors or social and environmental factors [47]. Therefore, parental behavior will need to be considered in future intervention studies.

An important finding of this study was that children who did not meet all three 24-h MG had a higher probability of overweight/obesity than those who met all the 24-h MG. Approximately 15% of the children in this sample were obese or overweight. This finding is consistent with the results of previous studies [48,49], and a previous systematic review examining the association between various combinations of 24-h movement behavior and obesity [16] reported that the optimum combination of high PA level, low screen time, and long sleep duration was associated with a lower risk of overweight/obesity compared to other combinations. Moreover, another study reported that compared to children who met all the 24-h MG, those who adhered to two of the guidelines were 2.6 times more likely to be obese/overweight, those who adhered to one of the guidelines were 4.7 times more likely to be overweight/obese, and those who did all not adhere to any of the guidelines were 8.2 times more likely to be overweight/obese [49]. Although it has been reported that it may be too early to detect adiposity in preschool-aged children with small inter-individual variability in the data [50], studies have demonstrated that obesity/overweight is associated with PA, screen time, and sleep, and our findings will have important implications as they support the theory that the three movement behaviors are interdependent. Since all the studies that have investigated the association between the 24-h MG and obesity to date have been cross-sectional, a causal association should be investigated in the future. Moreover, longitudinal and interventional studies are needed to quantify the effects of various combinations of 24-h MG on health outcomes such as obesity. In addition, it will be necessary to analyze the fat mass index and fat-free mass index using a measurement method for accurate obesity analysis.

Some limitations need to be considered in the interpretation of the findings in this study. First, with the cross-sectional design of the study, it was not possible to determine the causal association between adherence to the 24-h MG and the BMI z-score. For this, a longitudinal study or intervention study should be conducted to clarify such a relationship. Second, since this study focused on preschool children in the northeastern region of Japan, it is not clear whether the findings are generalizable for preschool children in all of Japan. Nevertheless, the results of this study provide strong support for integrated 24-h MG for Japanese preschool children, and since this is the first study of Japanese preschool children, the findings have important implications for the development of public health policies and effective intervention programs in the future.

## 5. Conclusions

In this study, only 21.5% of preschool children in Japan adhered to all three of the 24-h MG of the WHO. Most of the children adhered to the PA (91.6%) and sleep duration (82.5%) guidelines; however, fewer children adhered to the screen time guidelines (33.7%). Our study confirmed an association between adherence to the 24-h MG and the overweight/obesity status of preschool children. This finding has important implications as it supports the theory that the three-movement behaviors of PA, screen time, and sleep duration are interdependent. An increase in the prevalence of obesity and overweight is expected as children advance from the early years to adolescence, and, thus, the findings of this study are useful for establishing public health policies and intervention programs.

## Figures and Tables

**Table 1:** Descriptive characteristics of participants.

	Total Sample (*N* = 421) Mean ± SD	Boy (*N* = 216) Mean ± SD	Girls (*N* = 199) Mean ± SD	*p*-Value
Age (years)	4.62 ± 0.87	4.55 ± 0.85	4.7 ± 0.89	0.070
BMI (kg/m^2^)	15.65 ± 1.28	15.77 ±1.29	15.52 ± 1.26	0.039
BMI z-scores ^a^	0.14 ± 0.90	0.22 ± 0.96	0.05 ± 0.81	0.041
Moderate to vigorous physical activity (min/day)	98.65 ± 29.02	106.82 ± 28.92	90.31 ± 26.88	0.001
Total physical activity (min/day)	510.24 ± 104.72	517.37 ± 111.28	502.73 ± 97.04	0.152
Screen time (min/day)	118.59 ± 72.47	116.08 ± 66.67	120.91 ± 77.56	0.496
Sleep duration (h/day) ^b^	10.83 ±1.64	10.80 ±1.60	10.87 ± 1.67	0.713

Abbreviations: BMI body mass index, SD standard deviation. ^a^ BMI z-scores were calculated according to the World Health Organization (WHO) growth standards [27]. ^b^ Including nap(s) time.

**Table 2:** Associations between meeting the combinations of the 24-Hour Movement Guidelines and adiposity among young children (*n* = 421).

Meeting Recommendations	BMI z-Score	
	Unadjusted	Adjusted
	B (95% CI)	B (95% CI)
At least physical activity		
Meet	Reference	Reference
Do not meet	1.005 (0.840, 1.202)	1.001 (0.843. 1.187)
At lease screen time		
Meet	Reference	Reference
Do not meet	1.071 (0.960, 1.195)	1.056 (0.952, 1.172)
At least sleep duration		
Meet	Reference	Reference
Do not meet	1.151 (1.001, 1.332) *	1.148 (0.998, 1.323)
At least physical activity + screen time		
Meet	Reference	Reference
Do not meet	1.079 (0.964, 1.209)	1.030 (0.973, 1.091)
At least physical activity + sleep duration		
Meet	Reference	Reference
Do not meet	1.151 (0.997, 1.324)	1.148 (0.998, 1.323)
At least screen time + sleep duration		
Meet	Reference	Reference
Do not meet	1.124 (0.998, 1.263)	1.098 (0.983, 1.226)
Physical activity + screen time + sleep duration		
Meet	Reference	Reference
Do not meet	1.148 (1.020, 1.239) *	1.139 (1.009, 1.285) *

OR (95% CI): Odds ratio (95% confidence intervals). BMI z-scores were calculated according to the World Health Organization (WHO) growth standards; Adjusted analyses included children’s age, sex. Meeting the recommendations is defined as 180 min of total PA including more than 60 min/day of moderate-to-vigorous PA, no more than 60 min/day for screen time, and between 10 and 13 h/day for sleep duration. * *p* < 0.05.
